# Supplementary material for: Psychometric properties and longitudinal measurement invariance of the drug craving scale: Modification of the Polish version of the Penn Alcohol Craving Scale (PACS)
Source: PLoS One. 2021 Sep 8;16(9):e0256018. doi: 10.1371/journal.pone.0256018 (PMC8425527; doi:10.1371/journal.pone.0256018)
Supplement: S1 Appendix — (PDF) [file pone.0256018.s001.pdf]

S1 Appendix. The changes between the PDCS and the PACS positions content.

PENN **ALCOHOL** CRAVING SCALE (PACS)	PENN **DRUG** CRAVING SCALE (PDCS)
1. How often have you thought about **drinking** or about how good **a drink** would make you feel during this period?<br><br>0 never (0 times during this period of time)<br>1 rarely (1 to 2 times during this period of time)<br>2 occasionally (3 to 4 times during this period of time)<br>3 sometimes (5 to 10 times during this period of time)<br>4 often (11 to 20 times during this period of time)<br>5 most of the time (20 to 40 times during this period of time)<br>6 nearly all of the time (more than 40 times or more than 6 times per day)	1. How often have you thought about **taking drugs** or about how good **taking drugs** would make you feel during this period?<br><br>0 never (0 times during this period of time)<br>1 rarely (1 to 2 times during this period of time)<br>2 occasionally (3 to 4 times during this period of time)<br>3 sometimes (5 to 10 times during this period of time)<br>4 often (11 to 20 times during this period of time)<br>5 most of the time (20 to 40 times during this period of time)<br>6 nearly all of the time (more than 40 times or more than 6 times per day)
2. At its most severe point, how strong was your craving during this period?<br><br>0 none at all<br>1 slight, that is a very mild urge<br>2 mild urge<br>3 moderate urge<br>4 strong urge, but easily controlled<br>5 strong urge and difficult to control<br>6 strong urge and would have **drunk alcohol** if it were available	2. At its most severe point, how strong was your craving during this period?<br><br>0 none at all<br>1 slight, that is a very mild urge<br>2 mild urge<br>3 moderate urge<br>4 strong urge, but easily controlled<br>5 strong urge and difficult to control<br>6 strong urge and would **take drugs** if it were available
3. How much time have you spent thinking about **drinking** or about how good a **drink** would make you feel during this period?<br><br>0 none at all<br>1 less than 20 minutes<br>2 21-45 minutes<br>3 46-90 minutes<br>4 91 minutes - 3 hours<br>5 between 3 and 6 hours<br>6 more than 6 hours	3. How much time have you spent thinking about **taking drugs** or about how good **taking drugs** would make you feel during this period?<br><br>0 none at all<br>1 less than 20 minutes<br>2 21-45 minutes<br>3 46-90 minutes<br>4 91 minutes - 3 hours<br>5 between 3 and 6 hours<br>6 more than 6 hours

4. How difficult would it have been to resist **taking a drinking** during this period of time if you had known **a bottle** were in your house?<br>0 not difficult at all<br>1 very mildly difficult<br>2 mildly difficult<br>3 moderately difficult<br>4 very difficult<br>5 extremely difficult<br>6 would not be able to resist	4. How difficult would it have been to resist **taking drugs** during this period of time if you had known **the drugs** were in your house?<br>0 not difficult at all<br>1 very mildly difficult<br>2 mildly difficult<br>3 moderately difficult<br>4 very difficult<br>5 extremely difficult<br>6 would not be able to resist
5. Keeping in mind your responses to the previous questions, please rate your *overall average* **alcohol** craving for the stated period of time.<br>0 never thought about **drinking** and never had the urge **to drink**<br>1 rarely thought about **drinking** and rarely had the urge **to drink**<br>2 occasionally thought about **drinking** & occasionally had the urge **to drink**<br>3 sometimes thought about **drinking** & sometimes had the urge **to drink**<br>4 often thought about **drinking** & often had the urge **to drink**<br>5 thought about **drinking** most of the time & had the urge **to drink** most of the time<br>6 thought about **drinking** nearly all of the time & had the urge **to drink** nearly all of the time	5. Keeping in mind your responses to the previous questions, please rate your *overall average* **drug** craving for the stated period of time.<br>0 never thought about **drugs** and never had the urge **to use drugs**<br>1 rarely thought about **drugs** and rarely had the urge **to use drugs**<br>2 occasionally thought about **drugs** & occasionally had the urge **to use drugs**<br>3 sometimes thought about **drugs** & sometimes had the urge **to use drugs**<br>4 often thought about **drugs** & often had the urge **to use drugs**<br>5 thought about **drugs** most of the time & had the urge **to use drugs** most of the time<br>6 thought about **drugs** nearly all of the time & had the urge **to use drugs** nearly all of the time

PACS and PDCS © Research Society on Alcoholism
