# Supplementary material for: Psychometric properties and longitudinal measurement invariance of the drug craving scale: Modification of the Polish version of the Penn Alcohol Craving Scale (PACS)
Source: PLoS One. 2021 Sep 8;16(9):e0256018. doi: 10.1371/journal.pone.0256018 (PMC8425527; doi:10.1371/journal.pone.0256018)
Supplement: S2 Appendix — (PDF) [file pone.0256018.s002.pdf]

S2 Appendix. Polish version of the Penn Drug Craving Scale (PDCS).

SKALA GŁODU NARKOTYKU (PDCS)

opracowana na podstawie Penn Alcohol Craving Scale (PACS)[i] w polskiej adaptacji
J. Chodkiewicza, M. Ziółkowskiego, K. Gąsiora, A. Juczyńskiego, D. Czarneckiego, A. Biedrzyckiej, K. Nowakowskiej-Domagały

Proszę o uważne przeczytanie każdego z pięciu pytań i zaznaczenie przy każdym jednej cyfry, która najlepiej opisuje Pana/Pani głód narkotyku w ostatnim tygodniu

1. Jak często w ciągu ostatniego tygodnia myślałeś o zażywaniu narkotyku lub o tym, jak dobrze poczułbyś się po zażyciu?

    0 nigdy (ani razu w ciągu ostatniego tygodnia)
    1 rzadko (1 do 2 razy w ciągu ostatniego tygodnia)
    2 sporadycznie (3 do 4 razy w ciągu ostatniego tygodnia)
    3 czasem (5 do 10 razy w ciągu ostatniego tygodnia lub 1 do 2 razy dziennie)
    4 często (11 do 20 razy w ciągu ostatniego tygodnia lub 2 do 3 razy dziennie)
    5 przez większość czasu (20 do 40 razy w ciągu ostatniego tygodnia lub 3 do 6 razy dziennie)
    6 prawie przez cały czas (ponad 40 razy w ciągu ostatniego tygodnia lub ponad 6 razy dziennie)

2. Jak silny był Twój głód narkotyku w najgorszym momencie w ciągu ostatniego tygodnia?

    0 brak głodu
    1 słaby, czyli bardzo lekkie pragnienie
    2 lekkie pragnienie
    3 umiarkowane pragnienie
    4 silne pragnienie, ale łatwe do kontrolowania
    5 silne pragnienie i trudne do kontrolowania
    6 silne pragnienie i zażyłbym narkotyk, gdyby był dostępny

3. Ile czasu w ciągu ostatniego tygodnia spędziłeś na myśleniu o zażyciu narkotyku lub o tym, jak dobrze poczułbyś się po zażyciu?

    0 wcale
    1 mniej niż 20 minut
    2 21 do 45 minut
    3 46 do 90 minut
    4 91 minut do 3 godzin
    5 powyżej 3 do 6 godzin
    6 ponad 6 godzin

4. W ciągu ostatniego tygodnia jak trudno było by Ci się oprzeć przed zażyciem narkotyku, gdybyś wiedział, że masz w domu jego dawkę?

    0 zupełnie bez trudu
    1 z bardzo niewielką trudnością
    2 z niewielką trudnością
    3 z umiarkowaną trudnością
    4 bardzo trudno
    5 wyjątkowo trudno
    6 nie byłbym w stanie się oprzeć

5. Mając w pamięci swoje odpowiedzi na wcześniejsze pytania, oceń Twój ogólny przeciętny głód narkotyku w ciągu ostatniego tygodnia.

    0 wcale nie myślałem o narkotyku i nigdy nie odczuwałem pragnienia, by go zażyć
    1 rzadko myślałem o narkotyku i rzadko odczuwałem pragnienie, by go zażyć
    2 sporadycznie myślałem o narkotyku i sporadycznie odczuwałem pragnienie, by go zażyć
    3 czasem myślałem o narkotyku i czasem odczuwałem pragnienie, by go zażyć
    4 często myślałem o narkotyku i często odczuwałem pragnienie, by go zażyć
    5 przez większość czasu myślałem o narkotyku i przez większość czasu odczuwałem pragnienie, by go zażyć
    6 prawie cały czas myślałem o narkotyku i prawie cały czas odczuwałem pragnienie, by go zażyć

PACS and PDCS © Research Society on Alcoholism

[i] B. A. Flannery, J. R. Volpicelli, H. M. Pettinati, (1999). Psychometric Properties of the Penn Alcohol Craving Scale, *Alcoholism, Clinical and Experimental Research* 23, no. 8: 1289–95.
